# Pleural Effusions and Exploration of the Chest in Children

**Published:** 1907-02-02

**Authors:** 


A Journal of

The Medical Sciences and Hospital Administration.

Vol. XLI.—No. 1064., SATURDAY, FEBRUARY 2, 1907.

### Pleural Effusions and Exploration of the Chest in Children.

In a recent lecture at the Medical Graduates' College and Polyclinic, Dr. Geo. S. Middleton, of Glasgow, discussed some of the diagnostic aspects of pleural effusions, and as several of his conclusions are of considerable practical importance, attention may here be fittingly directed to them. The lecture was concerned more particularly with pleural effusions occurring in children, and it was pointed out that in such circumstances the clinical evidences of effusion may correspond very closely to those attending pulmonary consolidation. In other words, whilst deficiency or absence of the respiratory murmur is to be expected when there is a collection of fluid in the pleural cavity, it does sometimes happen, in such cases, that not only is the respiratory murmur distinctly heard over the dull area, but it has also a markedly tubular character, thus resembling the condition which exists in pulmonary consolidation. Whatever be the physical explanation, this is the clinical fact, and failure to remember it accounts for many mistakes in diagnosis.

When decision has once been taken that there is a collection of fluid in the pleura, the next question is: What is the nature of the fluid? And this usually means: Is the effusion serous or purulent? To provide an answer to this question the text-books advance a number of considerations which are said to incline the decision one way or the other; but Dr. Middleton boldly denies the validity of these, and states that there are no trustworthy signs in which confidence may be placed in the discussion between serous effusion on the one hand, and an empyema on the other. Quoting from his own experience, he finds an oscillating temperature, so often described as indicative of the presence of pus, to be not uncommon in cases of serous effusion, the presumed explanation being that such cases are of tubercular origin. As a parallel to this may be added the statement that a perfectly normal temperature, at least for a limited number of observations, is quite compatible with the presence of pus, even in considerable quantity, in the pleural cavity. Rigors and sweating are in exactly the same clinical position as a febrile temperature. Whilst often presented as proofs of the presence of pus, they, as a matter of fact, frequently accompany serous effusion, and, further, are often absent in cases of empyema. Another attempted distinction is found in the statement that whispered pectoriloquy over the dull area means serum, not pus, whilst with a purulent effusion brochophony may be present. Clinical experience does not support this statement, and Dr. Middleton evidently has not the slightest confidence in it. Thus all the usual clinical distinctions attempted to be established by means of physical examination between pleural effusion on the one hand and pulmonary consolidation on the other, are found to be untrustworthy and unreliable. Localised bulging, redness, or œdema of the chest wall, no doubt mean pus, but to allow the diagnosis to wait for one or other of these events is hardly a creditable proceeding, seeing that if pus is present prompt removal is an essential part of an efficient scheme of treatment.

It thus appears that in the child there may be difficulty in distinguishing between pneumonic consolidation and pleural effusion; and also in deciding in the case of effusion as to the nature of the fluid. When such doubt exists, Dr. Middleton urges very strongly that it should be resolved by the aid of the exploring needle. Especially is this necessary in children under two years of age, who "succumb comparatively rapidly to empyema and rarely recover unless operated on at an early date in the disease." Some few months ago an article was published conveying a grave warning of the dangers of exploratory puncture of the chest and quoting eleven cases in which this procedure was said to have been followed by death. No one will defend a practice which regards exploratory puncture of the chest as a trivial proceeding and one needing neither preparation nor consideration. The demand for it only arises when doubt exists and when all other attempts to resolve the doubt have been exhausted. But because in occasional instances carelessness, or some purely accidental circumstance, has led to disastrous results, that is no reason for surrounding with an atmosphere of alarm a practice which under proper conditions has been found to be perfectly safe. Further, if the use of the exploring needle has its responsibilities, neglect to use it carries a not less serious claim. For, as Dr. Middleton pertinently remarks, children sometimes die as cases of "pneumonia" whose lives might have been spared had the needle been used in time.

